# Surgical team communication and intraoperative errors: A cross-sectional study

**DOI:** 10.6026/973206300213214

**Published:** 2025-09-30

**Authors:** Naveenkumar Viswanathan, Sailesh Kumar, Soomal Jamil, Anvia Villina D'Souza, Alidjanov Xodijakbar Kashipovich

**Affiliations:** 1 Department of General Surgery, Prince Charles Hospital, Cwm Taf Morgannwg University Healthboard, Wales, United Kingdom; 2 Department of Surgery, University of Alabama, Birmingham, England; 3 Department of Forensic Medicine, Jinnah Medical and Dental college, Sharafabad, Karachi, 74800, Pakistan; 4 Department of General medicine, Madras Medical College, Chennai, Tamin Nadu, India; 5 Department of Faculty and Hospital, Tashkent Medical Academy, Tashkent, Uzbekistan

**Keywords:** Surgical outcomes, poor communication, intraoperative mistakes, safety, teamwork, operating room dynamics, cross-sectional research, human factors, surgical team conduct

## Abstract

The relationship between intraoperative error rates and the quality of communication within the surgical team is of interest. Using post-operative surveys and organized observation, 136 surgical procedures from various specializations were examined. The results showed that poor communication was strongly associated with higher error rates, especially when it came to important decision points and instrument handoffs. Real-time communication and effective preoperative briefings reduced the frequency of avoidable errors. Strengthening communication protocols may improve surgical safety and collaboration.

## Background:

Effective communication within the surgical team has a major impact on clinical outcomes and patient safety in the operating room (OR) [1]. Communication errors contribute to the occurrence of adverse events in various domains of health care [2]. High-stakes decision-making, interdisciplinary teamwork and complex surgical techniques create an environment highly vulnerable to communication-related errors [3]. Several studies have found that a significant proportion of adverse surgical events are caused by inadequate team communication, which includes misinterpretations, missing cues, and misunderstanding nonverbal signs [4]. Communication in the surgical field is influenced by a variety of factors, including team familiarity, surgical complexity, time constraints, and hierarchy [5]. Errors can happen in a variety of situations, including intraoperative choices, anesthetic updates, instrument swaps, and patient handovers [6]. The repercussions can range from minor technical problems to more significant problems including retained instruments, surgery performed at the incorrect place, or delays in emergency treatment [7]. Although the importance of communication in high-performance surgical teams has been demonstrated, most clinical settings continue to underuse standardized assessment and monitoring techniques [8]. Furthermore, the WHO Surgical Safety Checklist and other contemporary safety guidelines are frequently applied inconsistently [9]. The impact of communication dynamics on surgical errors in real-time practice must therefore be empirically evaluated immediately [10]. This cross-sectional study looks at the level of communication between surgical teams and how it relates to the severity of intraoperative errors [11]. Therefore, it is of interest to focus on observed and reported interactions across different specialties, the research intends to inform better communication training and system-level interventions that could enhance OR safety and efficiency.

## Materials and Methods:

This cross-sectional study was conducted over a 4-month period in the surgical departments of two tertiary care hospitals. A total of 136 elective surgical procedures were observed across general surgery, orthopedics, urology, and gynecology. The inclusion criteria were procedures lasting over one hour with at least three surgical team members present (surgeon, anesthesiologist, scrub nurse, and circulating nurse). Emergency surgeries were excluded to avoid confounding variables such as time pressure and incomplete team briefing. Trained clinical observers, who were not part of the surgical teams, used a validated communication behavior checklist to document instances of clear, ambiguous, or failed communication during each surgery. Key communication points analyzed included preoperative briefings, intraoperative instructions, instrument handoffs, and response to critical changes. In addition, post-operative debriefing surveys were administered anonymously to all team members to assess perceived communication quality and incident awareness. Intraoperative errors were defined as deviations from standard procedure, including wrong instrument use, incorrect site preparation, delayed response to vital sign changes, and breaches in sterility. These were logged and verified independently through surgical logs and observer reports. Demographic and procedural variables such as team composition, surgery duration, and team experience were also collected to control for confounding factors. Data were analyzed using descriptive statistics, chi-square tests, and logistic regression to determine the strength and significance of association between communication quality and intraoperative error rates. Ethical approval was granted by the institutional ethics committees, and informed consent was obtained from all surgical staff involved.

## Results:

Out of 136 surgeries observed, intraoperative errors were reported in 42 cases (30.9%). The analysis revealed a strong correlation between poor communication practices especially during instrument handoffs and response to complications and the occurrence of these errors. Surgeries involving teams with high communication scores had significantly fewer errors. Table 1 (see PDF) shows the distribution of surgical specialties observed. General surgery accounted for the largest proportion of cases (35.3%), followed by orthopedics (27.2%), urology (21.3%), and gynecology (16.2%). Table 2 (see PDF) depicts the frequency of intraoperative errors across specialties. General surgery contributed the highest proportion of errors (40.5%), followed by orthopedics (28.6%), urology (16.7%), and gynecology (14.2%). Table 3 (see PDF) differentiates the types of intraoperative errors observed (n = 42). The most common error was wrong instrument use (33.3%), followed by delayed response to patient vitals (23.8%), sterility breaches (21.4%), incorrect site preparation (11.9%), and missed verbal cues (9.6%). Table 4 (see PDF) compares surgeries with and without preoperative briefings. Procedures with structured briefings had a significantly lower error rate (13.9%) compared with those without briefings (50.0%). Table 5 (see PDF) shows the relationship between team experience and intraoperative error rates. Less experienced teams (<5 years) demonstrated higher error rates (47.2%) compared with moderately experienced (30.5%) and highly experienced teams (17.1%). Table 6 (see PDF) depicts the impact of communication breakdown frequency on errors. Surgeries with ≥5 breakdowns showed the highest error rate (69.0%), compared with 3-4 breakdowns (33.3%) and 0-2 breakdowns (13.2%). Table 7 (see PDF) differentiates error rates based on communication clarity during critical surgical events. Clear communication was associated with the lowest error rate (14.2%), while ambiguous (41.5%) and poor communication (77.7%) were linked to higher errors. Table 8 (see PDF) shows the relationship between instrument handoff accuracy and related errors. Frequent confusion led to the highest error rate (43.5%), compared with occasional delays (27.4%) and accurate/timely handoffs (8.2%). Table 9 (see PDF) depicts postoperative survey findings on team trust. Low trust was associated with a markedly higher error rate (80.0%), compared with moderate (33.3%) and high trust levels (11.5%). Table 10 (see PDF) compares logistic regression predictors of intraoperative errors. Poor communication clarity (OR = 5.71), absence of preoperative briefings (OR = 4.02), low team trust (OR = 3.88), and limited team experience (OR = 2.61) emerged as the strongest predictors.

## Discussion:

This cross-sectional survey identifies intraoperative communication as the key to avoiding surgical mistakes. There was a strong correlation between compromising communication habits namely, during handoffs and high-risk decision nodes and intraoperative error rates. Teams that did not use formal preoperative briefs had an error rate almost four times greater than those that did, emphasizing the safety benefit of systematic preparation. These results are consistent with previous research proving that standardized communication guidelines minimize preventable adverse events in the operating room [12]. Surgical communication is more than just voice. It involves coordination, psychological safety, team dynamics, and trust. Handoff errors on instruments were especially common, frequently due to rushed or vague communication, highlighting the importance of accurate and standardized handoff procedures. Notably, regression analysis identified that variables like procedure duration or case complexity were less predictive of intraoperative safety than preoperative briefings, team trust, and clear communication [13]. Practically, what the study implies is that surgical safety programs should first focus on interventions like closed-loop communication training, compulsory preoperative briefings, and simulation-based teamwork training. Promoting a culture of psychological safety is also critical, as this helps all members of the team--including junior staff --to raise concerns, hence minimizing communication breakdowns. The strong associations found in this study underpin integrating systematic communication assessments into regular surgical audits [14]. One of this research's greatest strengths is the complementarity of observational data in real time and postoperative questionnaires, yielding both objective and subjective accounts of team communication. Having multiple specialties increases generalizability as well. Yet a number of limitations must be noted. First, the observational nature restricts causal inference. Second, elective procedures only were studied, which may have underestimated error rates in emergency situations where time pressure is higher. Third, the presence of observers could have had some impact on team behavior (Hawthorne effect). Lastly, results are based on two tertiary hospitals that may not represent practices elsewhere in healthcare.

## Conclusion:

Ineffective intraoperative communication significantly raises the risk of surgical errors, particularly during critical instrument handoffs and decision-making. Two crucial protective factors are a high level of team trust and organized preoperative briefings. By reducing avoidable complications, streamlined communication protocols can increase surgical safety.
